# Improving predictive accuracy of a survey measure of risk for narcolepsy

**DOI:** 10.1080/21642850.2014.892430

**Published:** 2014-03-10

**Authors:** Jane F. Gaultney, David D. Gray, Kristin Daley

**Affiliations:** ^a^ Department of Psychology, University of North Carolina at Charlotte, Charlotte, NC, USA; ^b^ Charlotte Eye, Ear, Nose, and Throat Associates, Belmont, NC, USA

**Keywords:** narcolepsy, survey, screening instrument, Epworth Sleepiness Scale

## Abstract

Narcolepsy is a brain disorder that may go unrecognized and untreated for many years. The ability to use easily obtained survey information about symptoms of narcolepsy would facilitate identification of individuals potentially at risk for narcolepsy who could be referred for further testing. The purpose of the present study was to explore whether a survey instrument could successfully distinguish narcolepsy from other sleep disorders using data that could easily be obtained from a community or general patient sample. The hypothesized model added the Epworth Sleepiness Scale to a narcolepsy symptoms checklist to explore whether it improved accuracy of classification. Data related to symptoms were extracted from medical records of patients with a known diagnosis of narcolepsy, obstructive sleep apnea, or insomnia. The sample was then randomly split in half, allowing exploratory and confirmatory binary logistic regression. Adding the Epworth Sleepiness Scale score to the original list of symptoms more accurately classified those with or without narcolepsy. Although these findings require additional testing before they can be confirmed and generalized, they suggest that a self-report screening instrument for narcolepsy with acceptable accuracy is possible.

## Improving predictive accuracy of a survey measure of risk for narcolepsy

Diagnosis of many sleep disorders requires precise, detailed data such as that produced by polysomnography (PSG). Such sleep studies are expensive and may require an overnight stay in a sleep lab, making them impractical for widespread use as a screening tool. These diagnostic studies usually follow an initial clinical assessment to determine that such testing is justifiable. This requires the patient or a referring health care provider to recognize the need to initiate further sleep testing. If patients or healthcare workers do not recognize and correctly identify relevant symptoms, then the disorder may go undetected or misdiagnosed. Effective tools that can be used to quickly and inexpensively screen for sleep disorders could alert patients to the need to consider more involved testing. Such instruments could screen large samples in a wide variety of experimental and clinical settings. The current study proposed to improve the accuracy of a self-report measure to screen for narcolepsy.

Even with sophisticated and accurate measures of sleep, it can be difficult to diagnose narcolepsy. Narcolepsy is a neurological disorder that is associated with excessive daytime sleepiness (EDS), abnormalities of rapid eye movement (REM) sleep, muscle weakness with emotion (cataplexy), sleep paralysis, and hypnagogic hallucinations (Hale, 2010). All symptoms may not be present in all patients and may vary in intensity and frequency (Peacock & Benca, 2010). Because of difficulty in making a diagnosis of narcolepsy and delay in seeking treatment, the disorder is likely underdiagnosed and undertreated. One estimate suggested that as many as 50% of those with narcolepsy have not been correctly diagnosed (Hale, 2010). A survey of adults with narcolepsy in the UK indicated that delay from onset of symptoms to diagnosis ranged from 1 to 61 years, with an average lag of 10.5 years (Morrish, King, Smith, & Shneerson, 2004). Age of onset can be anywhere from childhood to middle adulthood, but one study found a mean of 23–24 years and greater frequency of onset at ages 14.7 and 35 (Dauvilliers et al., 2001). Patients with narcolepsy may present with greater daytime sleepiness than those with other sleep disorders (Doghramij, Lieberman, & Gordon, 2007); therefore, diagnostic evaluation usually includes a measure of daytime sleepiness, such as the multiple sleep latency test (MSLT) or Maintenance of Wakefulness Test. These tests are usually conducted at a medical facility, since they require controlled conditions and the use of electroencephalography, and are, therefore, not practical for large-scale or general-practice screening.

More recently, there have been increased cases of narcolepsy following the H1N1 vaccination, calling for an increased need for screening tools (Han et al., 2011). Although a diagnosis of narcolepsy cannot be determined by a survey instrument alone, an accurate screening tool could alert patients and health care professionals to the possibility of narcolepsy, perhaps increasing the chances that the disorder will be considered.

The use of self-reported data to screen for sleep disorders in large samples has been previously reported. For example, Hersberger et al. (2006) used survey data followed by individual counseling by a pharmacist to screen for several sleep disorders in Swiss pharmacies. Gaultney (2010) used a validated survey (Sleep-50; Spoormaker, Verbeek, van den Bout, & Klip, 2005) to screen for sleep disorders among college students. Conclusions from both studies, however, remain tentative since presence of a sleep disorder was not confirmed by clinical diagnosis. Other work, such as the validation studies for the Sleep-50 and the Sleep Disorders Questionnaire (Douglass et al., 1994), used survey measures to screen for sleep disorders and then compared the findings to already known diagnoses. The Sleep-50 adequately identified apnea, insomnia, restless legs/periodic limb movement disorder, circadian rhythm disorder, and nightmares, but did less well identifying narcolepsy. The Sleep Disorders Questionnaire reported scales for sleep apnea, narcolepsy, psychiatric sleep disorder, and periodic limb movements during sleep, of which the narcolepsy and sleep apnea scales were the most accurate.

The purpose of the present study was to explore whether data that could easily be obtained from a community sample could identify risk for narcolepsy by adding a sleepiness measure to a narcolepsy symptoms checklist. Sleepiness is a hallmark of, but not limited to, the disorder of narcolepsy. Other conditions (e.g. other sleep disorders, depression, and infection) are also associated with EDS. Individuals with narcolepsy, however, experience EDS most days, finding it difficult not to fall asleep during the day, especially in non-stimulating contexts (Morrison & Riha, 2012). Excessive sleepiness is often a presenting symptom, a starting point for diagnosis, and may be particularly high among patients with narcolepsy (Doghramij et al., 2007). We hypothesized that survey items, in combination with the Epworth Sleepiness Scale, could accurately distinguish patients with narcolepsy from those with another sleep disorder.

## Methods

### Participants

Data on 172 patients (45% male, *M*_age_ = 43.19, SD_age_ = 14.32; *M*_BMI_ = 30.06, SD_BMI_ = 8.25; 45 narcolepsy only, 19 narcolepsy plus insomnia, three narcolepsy plus obstructive sleep apnea [OSA], one diagnosed with all three disorders, and 104 with insomnia and/or OSA but not narcolepsy) were extracted from the charts that spanned a two-year period (2009–2011) at a large sleep medicine practice in the southeastern USA. Inclusion criteria were a diagnosis of OSA, insomnia, or narcolepsy and an available Epworth Sleepiness Scale score and/or (for those diagnosed with narcolepsy) an MSLT score.

Narcolepsy, insomnia, and OSA were diagnosed using the International Classification of Sleep Disorders 2 (American Academy of Sleep Medicine, 2005) definitions. All cases of insomnia included in this sample were diagnosed with psychophysiological insomnia. Hypocretin deficiency in patients with narcolepsy was not routinely measured at the time of diagnosis. Cataplexy was based on self-report or, in some cases, family interview. Patients with OSA or insomnia were limited to those who had been diagnosed around the same time as the patients with narcolepsy and for whom an Epworth Sleepiness Scale score was available. Thirteen of the patients diagnosed with narcolepsy (19%; 8% of total sample) had no Epworth Sleepiness Scale score in their medical charts. Comparison of narcolepsy patients with vs. those without Epworth Sleepiness Scale scores indicated no significant differences in age, *t*(66) = .42, *p* = .68, gender, or symptoms of narcolepsy, *χ* ^2^s(1) ≤ 2.46, *p*s ≥ .12. Since the Epworth Sleepiness Scale scores did not appear to be missing systematically for symptoms of narcolepsy nor for degree of daytime sleepiness, the missing scores were imputed using linear interpolation in order to avoid potential bias due to listwise deletion of cases (Roth, 1994).

The sample of narcolepsy patients was unusual in that only 19% reported cataplexy, whereas the literature suggests that the number should be higher (Doghramij et al., 2007). This may be explained by the practice's use of REM onset < 15 minutes as a defining feature of narcolepsy. It is possible use of this criterion led to greater identification of patients with narcolepsy in the absence of cataplexy. A preliminary analysis indicated that the model was a better predictor of narcolepsy with cataplexy. However, because the sample size of narcolepsy with cataplexy was small, no analyses comparing classification accuracy for those with or without cataplexy were reported here.

### Materials

Using the items from the narcolepsy scale in the Sleep-50 as a guide, a checklist of symptoms of narcolepsy and daytime impact were compiled. The Sleep-50 was developed by Spoormaker et al. (2005) and validated against clinical diagnoses that included PSG and/or MSLT (considered as “gold standards” for diagnosing some sleep disorders and daytime sleepiness, respectively) as clinically indicated. The survey items were scored to produce scales for narcolepsy and several other sleep disorders, as well as a scale for daytime impact. In this validation study, the narcolepsy scale had a specificity score of .86 and a sensitivity score of .67.

We extracted patient-reported symptoms of narcolepsy (hypnagogic hallucinations, paralysis, cataplexy, daytime sleep “attacks”, and fall asleep on social occasions) and daytime impact (tired at getting up, daytime sleepiness, lack of energy, easily irritated, difficulty concentrating, worry that I do not get enough sleep, and generally sleep badly) from patient charts. The original survey items from the Sleep-50 asked participants to indicate how true each symptom was for them using a scale of 1 (not at all true) to 4 (very much true). Data in the medical charts were available only in the form of yes/no answers, so each symptom was coded as present or not. While analyzing yes/no responses rather than the four-point scale used by the Sleep-50 was not ideal, examination of the item loadings reported in the Sleep-50 validation study suggested that binary responses might behave in a similar manner as the four-point scale. In the validation study, symptoms of narcolepsy loaded exclusively on the narcolepsy or daytime impact scales (both of which are used to identify risk for narcolepsy) with one exception. Cataplexy also loaded on the apnea scale (.32), although it was more strongly associated with narcolepsy (.47).

The Epworth Sleepiness Scale (Johns, 1991) consists of eight items indicating how likely the respondent is to fall asleep in various situations (e.g. sitting at a stoplight, after lunch). Each item is scored using a scale from 0 (would never doze) to 3 (high chance of dozing). The responses are summed. Higher scores indicate greater daytime sleepiness. A score > 10 indicates sleepiness of clinical interest and a score ≥ 16 indicates a dangerous level of sleepiness, usually found in individuals with moderate to severe OSA, hypersomnia, or narcolepsy (Johns, 1991). The scale has been widely reported in the literature and has good sensitivity and specificity for narcolepsy (Johns, 2000). Only the summed score was included in the medical charts; the specific items were not available. We used the Epworth Sleepiness Scale score in our calculations rather than the more definitive MSLT because of our goal to use data that could be easily collected in a variety of settings. The Epworth Sleepiness score can easily fit into a survey format for large-scale or general-practice screening.

### Procedure

An institutional review board at a large state university in the southeastern USA reviewed the protocol and certified that the project was exempt from oversight under category four. Experimenters did not have direct contact with patients. We extracted self-reported data from medical charts of patients from the sleep practice, recording the symptoms of narcolepsy, daytime impact, an MSLT score (for patients with narcolepsy), clinical diagnosis (made by a board-qualified sleep physician), Epworth Sleepiness Scale Sleepiness scores, and demographic information. The chart data were collected as part of the intake procedure for new patients; all reports reflected symptoms prior to diagnosis and treatment. Participants were identified through the sleep center patient database by the final diagnosis code. Identifying information was not recorded in the data file.

### Analyses

Patients were randomly selected for inclusion in one of two samples (exploratory and confirmatory; Tukey, 1980; see Table 1 for descriptive information about the two samples). Analyses and descriptive data involving the Epworth Sleepiness score reported here include the interpolated values. Data from the first group (*n* = 86) were analyzed to explore a binary logistic regression model that included the predictors of narcolepsy and daytime impact in binary form (block 1). A second block tested the hypothesized model by adding an Epworth Sleepiness score. The analyses were repeated with the second sample (*n* = 86) to confirm the initial results. The outcome variable was the presence (alone or in combination with another disorder) or absence of a diagnosis of narcolepsy. In each case, the statistic of interest was the model's rate of successful classification. This type of regression analysis reports accuracy of classification as would a discriminant analysis. Because discriminant analysis makes a number of assumptions, including normal distribution of predictors and equal variance, logistic regression is a preferred method by some to determine the success of classification (Tabachnick & Fidell, 1996).

**Table 1.** Demographic and descriptive data.

First Sample ( = 86)	% (*N*)	*M* (SD)	Range
Gender (% male)	46 (40)		
Narcolepsy	42 (36)		
Hypnagogic hallucinations	17 (30)		
Fall asleep in social occasions	1 (2)		
Daytime sleep “attacks”	9 (16)		
Cataplexy	8 (13)		
Sleep paralysis	15 (26)		
Daytime impact		1.64 (0.85)	0–6
Age		46.60 (14.61)	19–58
Epworth Sleepiness Scale^a^		13.70 (5.11)	3–23
Second sample (*N* = 86)			
Gender (% male)	44 (38)		
Narcolepsy	37 (32)		
Hypnagogic hallucinations	16 (14)		
Fall asleep in social occasions	1 (1)		
Daytime sleep “attacks”	9 (8)		
Cataplexy	8 (7)		
Sleep paralysis	14 (12)		
Daytime impact		1.71 (0.91)	0–6
Age		43.78 (14.08)	15–79
Epworth Sleepiness Scale^a^		13.42 (4.84)	2–24

^a^Includes interpolated data.

## Results

In each sample, we tested two blocks of predictors: (1) symptoms of narcolepsy and daytime impact and (2) these items plus an Epworth Sleepiness Scale score. See Table 2 for the results of the binary logistic regressions. The patterns of results were similar in both the exploratory and confirmatory samples.

**Table 2.** Exploratory and confirmatory binary regression analyses.

	Nagelkerke				
	*R*^2^	*χ*^2^	*χ*^2^Δ	Specificity^a^	Sensitivity^b^
Exploratory (*n* = 86)					
Block 1	.72	66.46**	–	96	78
Block 2	.78	75.12**	8.65**	96	81
Confirmatory (*n* = 86)					
Block 1	.65	55.89**	–	100	66
Block 2	.80	76.50**	20.61**	94	84

Notes: *Block 1*: hypnogogic hallucinations, cataplexy, sleep paralysis, fall asleep social occasions, daytime sleep attacks, tired at awakening, sleepy during day, like more energy, easily irritated, difficulty concentrating, worry not enough sleep, and generally sleep badly. *Block 2*: hypnogogic hallucinations, cataplexy, sleep paralysis, fall asleep social occasions, daytime sleep attacks, tired at awakening, sleepy during day, like more energy, easily irritated, difficulty concentrating, worry not enough sleep, generally sleep badly, and Epworth Sleepiness Scale.

^a^Percent of patients who DID NOT have narcolepsy who were correctly excluded.

^b^Percent of patients who DID have narcolepsy who were correctly included.

**p<.01.

As was the case in the Sleep-50 validation study, symptoms of narcolepsy and daytime impact did an excellent job of excluding those who did not have narcolepsy (specificity in block 1 ≥ 96% in both samples). Block 1 items had lower sensitivity (≥66%) similar to that found by Spoormaker et al. (2005). Adding an Epworth Sleepiness Scale score in a second block to the model did not change specificity in the exploratory sample and lowered it by 6% in the confirmatory sample; however, sensitivity significantly improved in both samples. Overall accuracy of classification was slightly higher for the confirmatory group.

## Discussion

The goal of the study was to determine whether easily obtained screening questions could accurately include patients with a known diagnosis of narcolepsy and exclude patients without such a diagnosis. Data from a screening instrument would not be intended to take the place of accepted diagnostic tests, but rather to identify those who might benefit from further investigation of symptoms of narcolepsy. As hypothesized, adding the Epworth Sleepiness Scale score to a list of symptoms of narcolepsy and daytime impact produced more accurate classification within a clinical, adult population.

The Epworth Sleepiness Scale score explained significant additional variance even after accounting for daytime impact. This pattern was true for both the exploratory and confirmatory samples. Although the daytime impact and sleepiness constructs measured by these variables are interrelated, the Epworth Sleepiness Scale appears to measure something about risk for narcolepsy not already tapped by the daytime impact score. The two measures may reflect different levels of specificity. The daytime impact scale included indicators of perceived sleepiness (tired at getting up and daytime sleepiness), fatigue (lack of energy), sleep quality (generally sleep badly), as well as cognitive or emotional outcomes (easily irritated, difficulty concentrating, and worry about getting enough sleep). The Epworth Sleepiness Scale asks about the likelihood of falling asleep in various common situations. Therefore, it may be a more specific measure of the perceived sleepiness that is a characteristic of narcolepsy.

Sensitivity improved in the present data after adding the Epworth Sleepiness Scale, while specificity remained high. As was true in the original validation study (Spoormaker et al., 2005), the tested models did a better job at correct exclusions than correct inclusions, which should underestimate prevalence of narcolepsy. This is contradictory to the findings of Gaultney (2010), who found that the original scale appeared to overestimate prevalence of narcolepsy in a community sample of college students. The college community sample, however, was younger than most participants in the present sample. Furthermore, college students may have been excessively sleepy for reasons not related to a sleep disorder and may have over-reported symptoms of sleep disorders (Uher et al., 2009).

An advantage of the present data is that participants' diagnoses had been established by a board-certified sleep physician following established diagnostic procedure; therefore, there was reasonable confidence that the diagnoses were correct. However, these data can be generalized only to a clinical population of adults with narcolepsy, insomnia, or OSA. It did not establish whether the findings generalize to individuals in a community sample, or within a younger age group. In order to conclude that adding the Epworth Sleepiness Scale to the Sleep-50 narcolepsy scale will improve accuracy in diverse samples, a prospective study followed by professional diagnosis of those at risk in a community sample is needed. These data, therefore, are simply a first step in refining an effective screening tool. Although it seems very likely that adding the Epworth Sleepiness Scale will improve accuracy when identifying individuals who might benefit from further investigation into the risk for narcolepsy (Johns, 1991), this finding needs verification.

The goal of the present study was to improve a survey measure of risk for narcolepsy using measures suitable for large, community samples. The Epworth Sleepiness Scale is widely available, easily administered, and a reasonably good estimate of daytime sleepiness (Johns, 1991). If future research establishes that its inclusion consistently improves the accuracy of classification of narcolepsy risk within various populations, then it could easily be added to an existing survey measure. For example, the criteria for designation of “at risk for narcolepsy” in the Sleep-50 could be modified so that minimum scores for the narcolepsy scale, the daytime impact scale, *and* the Epworth Sleepiness Scale score must be exceeded in order to classify an individual as at risk for narcolepsy. The clinical data reported here did not use the four-point scale from the Sleep-50 survey, however, preventing direct comparisons. Although the Sleep-50 rated symptoms on a four-point scale, while the present data used a binary format, the similarity of these results to those of the Sleep-50 validation study suggests that the use of binary responses functioned in a similar manner to the four-point scale.

A survey screening tool would need to establish an optimal cut-off score on the Epworth Sleepiness Scale to distinguish narcolepsy from other sleep disorders. Other research suggests that a cut-off ≥ 16 may be needed (Doghramij et al., 2007; Johns, 1991; Mahmoudi, Won, & Mohsenin, 2013), but this remains to be established.

Improving the accuracy of self-reported data identifying risk for narcolepsy may be useful in both research and clinical settings, including clinical settings that are not focused primarily on sleep. The ability to screen with reasonable accuracy for the disorder may identify symptoms that would otherwise go unidentified, untreated, or improperly treated. Although only a small percentage of adults have narcolepsy (Longstreth et al., 2007), there have been recent incidences of “outbreaks” of narcolepsy related to the elimination of hypocretin post H1N1 vaccination (Han et al., 2011). Affected individuals might not be seen by sleep specialists in the normal course of events. Although such a screening tool would never be a replacement for comprehensive diagnostic process, an easily available screening instrument could allow for appropriate patient selection for more involved testing.
